# Effects of non-pharmaceutical interventions on social distancing during the COVID-19 pandemic: Evidence from the 27 Brazilian states

**DOI:** 10.1371/journal.pone.0265346

**Published:** 2022-03-17

**Authors:** Rodrigo Fracalossi de Moraes, Louise B. Russell, Lara Livia Santos da Silva, Cristiana M. Toscano

**Affiliations:** 1 Institute for Applied Economic Research (Ipea), Rio de Janeiro, Brasil; 2 Department of Medical Ethics and Health Policy, Perelman School of Medicine, and Leonard Davis Institute of Health Economics, University of Pennsylvania, Philadelphia, Pennsylvania, United States of America; 3 Department of Collective Health, Institute of Tropical Pathology and Public Health, Federal University of Goiás, Goiânia, Goiás, Brasil; Universidad Nacional Autonoma de Nicaragua Leon, NICARAGUA

## Abstract

**Background:**

Despite substantial evidence on the effectiveness of non-pharmaceutical interventions (NPIs), there is still limited evidence on the individual effects of different types of NPIs on social distancing, especially in low- and middle-income countries.

**Methods:**

We used panel data analysis to evaluate the effects of mandatory social distancing rules on social distancing. We obtained data on six different categories of mandatory restrictions implemented in Brazil, by date and state, from state government gazettes (*diários oficiais*). We then defined a social distancing rules index (SDI) to measure the strictness of social distancing rules, assigning each a value of 2, 1, or 0 depending on whether restrictions were full, partial, or very limited/non-existent at every given time. A separate variable was defined for masking mandates. We tested whether the following variables were associated to social distancing: SDI, masking mandates, COVID-19 incidence, population socioeconomic status, and political orientation. Data is for each day between March 11^th^ and November 10^th^, 2020 in the 27 Brazilian states (*N* = 6615).

**Findings:**

Social distancing increased when social distancing rules were stricter, and decreased when the use of face masks became mandatory. The effects of different types of restrictions varied: suspending in-person classes and gatherings, religious/sport/cultural activities had a greater effect than other types of restrictions. Also, the effect of social distancing rules on people’s behaviour decreased over time, especially when rules were stricter.

**Interpretation:**

Mandatory social distancing rules must be adopted to increase social distancing. Stricter rules have a higher impact, but result in decreased compliance over time. Policymakers should prioritize more targeted policies.

## Introduction

In the absence of widespread vaccination or treatments against COVID-19, social distancing is strongly recommended to reduce infection transmission rates [1–5]. The practice of social distancing means staying home or at least away from others as much as possible to help prevent spread of the disease in the community. By minimizing physical contact between people, transmission of infection is reduced, particularly for COVID-19, which is transmitted mainly through respiratory droplets. As such, social distancing measures have been widely adopted to mitigate the COVID-19 pandemic.

Yet, what determines levels of social distancing in a society? Should governments expect people to practice social distancing voluntarily or should they adopt mandatory measures? If mandatory measures are adopted, for how long are people likely to comply with them before having ‘pandemic fatigue’? Which types of NPIs are the most effective in making people stay at home? Answering these questions is important for both public health and economic reasons. Strict social distancing measures reduce transmission rates but slow down economies [6,7]. They may also have distributive and intergenerational effects since socioeconomic impacts are stronger on the most vulnerable and there are potential effects on public indebtedness [8]. These effects are especially severe in low- and middle-income countries, where a high proportion of the population works in the informal sector [9,10] and governments have lower fiscal capacity, which is essential for stimulating the economy.

In the early stages of the pandemic, some evidence suggested that people practiced social distancing voluntarily, and that this effect was stronger than the effect of mandatory restrictions in all but the poorest countries [11]. Case studies in the United States and Sweden seemed to reinforce this finding as people adopted social distancing *before* mandatory restrictions were introduced. In addition to fear of getting infected, this behaviour would have been driven by empathy for others [12], trust in science [13,14], or trust in government institutions [15].

However, NPIs in the form of mandatory social distancing rules significantly increased the likelihood of someone staying at home [13,16–20]. The effect of these NPIs on people’s behaviour is not constant across time and space though, nor among different individuals. A study in the United States demonstrated that political conservatism predicted less compliance with behaviours aimed at preventing spread of COVID-19 [21]. The widespread circulation of conspiracy theories also influenced people’s behaviour. In the United States, people holding more conspiracy beliefs at the beginning of the pandemic showed the lowest increase in social distancing [22]. Mandatory rules were also associated with individual traits: people with traits associated to anxiety, prosociality, and rule compliance were more likely to practice social distancing [23]. The duration of restrictions is also likely to influence social distancing levels: the longer the mandatory restrictions were in effect the less people complied with them [20]. Frequent extensions can create confusion and frustration, making people less likely to comply with rules–as observed in Italy [24]. This is important for policymakers because the *timing* of social distancing rules may indirectly influence transmission rates: if adopted too early, compliance might be low later on when most needed.

Moreover, different types of NPIs are likely to have different effects on social distancing levels and, as a consequence, on transmission rates. An analysis of different NPIs found that suspending gatherings, closing schools and imposing border restrictions were the most effective measures in reducing transmission [25], but the data covered only the months of March and April 2020, raising questions about people’s behaviour over longer periods of time. Looking only at March–April 2020 is also limited because social distancing measures were often adopted simultaneously in various countries, making it hard to isolate the individual effects of different types of restrictions. In addition, the impacts of different types of NPIs may be drastically different depending on the country adopting them and when they were adopted [25].

This paper examines the determinants of social distancing levels during the COVID-19 pandemic in Brazil. Brazil provides an opportunity to investigate the determinants of social distancing levels because policies were implemented primarily by state governments, with no coordination by the national government. As such, it enables us to compare state policies and outcomes within the same country while controlling for factors that are hard to hold constant in cross-country comparisons. In this article, focus is on the effects of mandatory social distancing rules on people’s behaviour and on the interaction between the strictness of restrictions and their duration. We also examine the role of masking.

## Materials and methods

We conducted a balanced panel data regression analysis using daily state-level data for the period from March 11^th^, 2020 to November 10^th^, 2020. The study period starts when the WHO declared the global COVID-19 pandemic–which prompted various governments to adopt social distancing measures–and includes eight months, encompassing the whole first wave of the pandemic in Brazil. All the 27 Brazilian states were included in the analyses (Distrito Federal was considered a state). We tested the following hypotheses:

*H1*. *The stricter the mandatory social distancing rules the more people practiced social distancing*.
*H2*. *Restricting certain activities had a greater impact on increasing social distancing than restricting others*.
*H3*. *As the duration of mandatory social distancing rules increased*, *compliance decreased*.
*H4*. *The stricter the social distancing rules the faster social distancing levels decreased over time*.
*H5*. *Mandatory masking decreased social distancing*.
*H6*. *The higher the incidence of new COVID-19 cases the more people practiced social distancing*.
*H7*. *The lower the socioeconomic level of a state the less people practiced social distancing*.
*H8*. *The higher the proportion of right-wing voters in a state the lower the social distancing levels*.

### Dependent variable

#### Social distancing levels

The dependent variable is the level of social distancing. The unit of analysis is the state-day, considering the 27 Brazilian states over the analysed period. Social distancing levels are inversely related to mobility: the more people stayed at home in a given day the higher the social distancing levels. We therefore used data from the Brazilian geolocation company In Loco (later renamed Incognia), which collected data about daily levels of social mobility through apps in over 60 million smartphones in Brazil–similar to Google Mobility Reports. In Loco used various apps, including those of major telecommunication companies, retail stores, and banks [26], aggregating the data into the ‘social distancing index’, used here as a measure of social distancing, which has been done in previous research [27,28]. This index is expressed in percentages where 100% means that everybody (in the sample) stayed at home for a whole day in a given geographical area.

### Independent variables

#### 1. Strictness of mandatory social distancing rules

To measure the strictness of social distancing rules we use the social distancing rules index (SDI), introduced in previous work [29] and used elsewhere [20,30–32]. This index measures whether the following activities or places were restricted or suspended at every given time: 1) gatherings, cultural, sport and religious activities; 2) non-essential shops and offices; 3) restaurants, bars, pubs, and similar places; 4) non-essential industries; 5) schools; and 6) public transportation. Each measure was assigned a value of 2, 1 or 0 depending on whether suspension or restriction was full, partial, or very limited/non-existent–and the index is the sum of these values. The index’s values were adjusted to be between 0 and 10 (a more intuitive scale than 0 to 12), in which 10 is the greatest level of restriction. Information on implemented measures came from state legislation published in the 27 state government gazettes (*diários oficiais*). Details of the criteria used for coding each of these variables are described in the supplemental appendices (S1 Table). The data used in this article are available at Harvard Dataverse [33]. Data on social distancing measures in Brazil covering a longer period are available at https://medidas-covidbr-iptsp.shinyapps.io/painel and http://tinyurl.com/ipeacoronavirus.

#### 2. Incidence of new cases of COVID-19

We hypothesized that a growing incidence of cases should increase awareness about the epidemic and consequentially people’s fear of getting infected or infecting others, and as such influence their behaviour. We used rolling averages (considering the seven days between *t* − 6 and *t*) of the number of new COVID-19 cases per 100,000 people. Data was obtained from the Brazilian Ministry of Health [34]. Underreporting of cases could be a problem, but there was a high correlation between the numbers of new cases and new deaths, suggesting that underreporting rates did not vary substantially over time: the Pearson correlation coefficient between rolling averages of new cases and new deaths between March 11^th^ and November 10^th^, 2020 was above (or close to) 0.6 for the 27 states, all statistically significant at a confidence level of 95%.

While rising incidence of new cases is hypothesized to lead to stricter social distancing measures, it is also true that higher levels of social distancing are expected to lead to a subsequent decline in incidence of new cases. The relationship runs only forward in time: new cases at time *t* or earlier cause social distancing at time *t* and later, but social distancing at time *t* and later does not cause cases earlier in time. We therefore included incidence at time *t* in the regressions to explain social distancing at time *t*. Our incidence measure incorporates a lag between incidence of new cases and social distancing because the rolling average at *t* includes 6 days before as well as day *t* and because reported cases at *t* lag actual cases at *t*. We experimented with longer lags, lagging incidence one and two weeks, but the results were the same so we report the results for incidence at *t*.

#### 3. Duration of social distancing rules

The longer the rules last the less likely people are to comply with them: social isolation causes psychological fatigue; people may seek to escape from domestic abuse; or people might have to work once savings are exhausted. Duration of rules is measured by the number of days since the first mandatory social distancing rule was introduced in a state. For example, if mandatory restrictions on social and economic activities (of any type) were in place for 30 days, the value of this variable for that day is 30. Values for days when there was not any measure in place are 0. All states changed the strictness of rules over the analysed period, but rules were never completely dropped. So, this variable amounts to a linear time trend which starts with the first NPI.

The duration of rules should also interact with the strictness of mandatory social distancing rules. Strict rules should be unlikely to be followed for long periods of time, but less strict rules should be more tolerable. Levels of compliance should therefore decrease faster when rules are stricter.

#### 4. Socio-economic levels

Negative impacts of the epidemic and of mandatory social distancing rules are proportionally higher on the poor [8,35]. In poorer places, people should be less likely to practice social distancing because they are less likely to have savings and more likely to have informal jobs. Moreover, poverty is highly correlated with years of education, which might negatively influence people’s ability to understand information about the pandemic, especially in a context in which the national government and other actors in Brazil systematically disseminated disinformation. Socioeconomic level is measured by GDP per capita in each state in 2018 (data is from the Brazilian Institute of Geography and Statistics–IBGE) [36]. Values of this variable are fixed over the analytical period.

#### 5. Political orientation of voters

The political orientation of people might influence their willingness to practice social distancing. A stronger sense of social responsibility should be more common among left-wing people. In contrast, those who vote for right-wing candidates should put more emphasis on their freedom of movement. In the models presented in this article, this is measured by the proportion of people who voted for Brazil’s current president Jair Bolsonaro (a far-right politician) in the run-off of the 2018 presidential election, being fixed for the analytical period. Data for each of the states was obtained from Brazil’s Superior Electoral Court (*Tribunal Superior Eleitoral*–TSE) [37].

#### 6. Mandatory masking

Once wearing a mask becomes mandatory, people’s feeling of safety should increase because they are less likely to get infected or transmit infection to others. We created two dummy variables. One of them is for partial masking, which are cases when masking was mandatory in some places or under certain circumstances (e.g., in shops, public transportation, churches). The other is for full masking, which are cases when face masks were mandatory in both public and private spaces (except in someone’s house or private cars). No mandatory masking is the reference category, which is for cases when the use of masks was not mandatory, or was mandatory only for a limited number of people or circumstances (e.g., for health workers). From the 19^th^ August 2020 on, the value of 2 was assigned to all states because wearing a face mask in public and private spaces became mandatory in the whole country and remained so until the end of the period analysed. Information for this variable came from state legislation published in the 27 state government gazettes (*diários oficiais*).

### Statistical analysis

Initially, we looked at the strictness of the social distancing rules and social distancing levels in the 27 Brazilian states, contrasting these geographically. We then evaluated the correlations between the strictness of social distancing rules and levels of social distancing in all states, further detailed in the supplemental appendices (S1 Fig). Their Pearson correlation coefficients were above 0.5 in 23 states (and 0.43, 0.29, 0.27 and 0.20 in the other four states).

Next, we did a panel data analysis (using Stata 13), with daily data for all 27 states from March 11^th^ to November 10^th^, 2020, totalling 6,615 data points. We ran six models considering different combinations of the following variables: 1) strictness of mandatory social distancing rules (SDI); 2) incidence of new cases of COVID-19; 3) duration of social distancing rules; 4) socio-economic levels; 5) political orientation of voters; and 6) mandatory masking. Initially, we included all independent variables (model 1). In this model, state dummies were not included as they were likely to capture the effect of a few of the covariates.

As the variables ‘political orientation of voters’ and ‘socio-economic levels’ were highly correlated (*r* = .62), we ran separate models (models 2 and 3) including in each only one of these variables, thus preventing potential bias from multicollinearity. As there was also a high correlation (*r* = .73) between the variables ‘duration of social distancing rules’ and ‘mandatory use of face masks’, we also ran separate models (models 4 and 5) with only one of these variables (including also state dummies). Model 6 included only the variables which were significant (*p*-value < 0.05) in models 1–5, including also state dummies and an interaction between the strictness and duration of social distancing rules. Eq 1 below presents the specification of model 6.

$$\begin{array}{l} {SD}_{it}=\beta_{0}+\beta_{1}({SDI}_{it})+\beta_{2}({Duration}_{it})+\beta_{3}({MaskPartial}_{it})\\ +\beta_{4}{(MaskFull}_{it})+\beta_{5}({SDI}_{it}\times {Duration}_{it})+\alpha_{i}+u_{it} \end{array}$$ (1)

where *SD*_*it*_ is the level of social distancing expected in Brazilian state *i* at day *t* (in a scale of 0–100%), *SDI*_*it*_ is the strictness of mandatory social distancing measures in Brazilian state *i* at day *t* (in a scale of 0–10), *Duration*_*it*_ is the number of days (at day *t*) since the first mandatory social distancing measure was adopted in Brazilian state *i*, *MaskPartial*_*it*_ and *MaskFull*_*it*_ are dummy variables measuring whether there was mandatory masking (partial and full, respectively) in Brazilian state *i* at day *t*, α_*i*_ is the dummy variable for state *i*, and *u*_*it*_ is the error term.

Next, we ran three models estimating the individual effects of six different types of social distancing rules. In models 7–9 the variable *SDI*_*it*_ was replaced by the level of strictness (with values of 0, 1 or 2) of each one of the six types of NPIs considered in the analyses. Models 8–9 include either ‘duration of social distancing rules’ or ‘mandatory use of face masks’ given their high correlation. The variables ‘political orientation of voters’ and ‘socio-economic levels’ were not included because they were likely to be captured by the state dummies.

In all models, we added a dummy variable for weekends and bank holidays since the proportion of people staying at home during these days is likely to be higher. We used cluster-robust standard errors in all models so that standard errors are robust to serial correlation and heteroskedasticity.

## Results

There was not much variation across Brazilian states regarding the date of implementation of the first mandatory NPIs. The first state to implement them was Distrito Federal on March 11^th^, 2020 and the last ones were Mato Grosso do Sul and Rio Grande do Sul on March 19^th^, 2020. Yet, there was substantial variation in the strictness of NPIs across states and over time, with rules being the strictest from late March through April 2020. The same applies to social distancing levels, which were higher during the first weeks of mandatory social distancing rules. Table 1 shows data for these two variables for all Brazilian states, comparing their averages in the first two months of the analysed period (11 March– 10 May 2020) to those of the rest of the period (11 May 2020–10 November 2020). Data are also presented graphically in the supplemental appendices (S1 and S2 Figs). Table 1 also presents data for other variables analysed in this article.

**Table 1:** Measures of social distancing, strictness of social distancing rules and other variables, by state, Brazil.

State	Social distancing levels, 0–100% (average per period)			Strictness of social distancing rules, 0–10 (average per period)			GDP per capita (Brazilian Reais, 2018)	Percentage of votes for Bolsonaro (run-off 2018 elections), 0–100%	Introduction of full mandatory masking
	11 Mar-10 May 2020	11 May-10 Nov 2020	*t*(243), *p*-value	11 Mar-10 May 2020	11 May-10 Nov 2020	*t*(243), *p*-value			
AC	48.0	42.8	6.8, < .001	6.1	4.7	5.2, < .001	17,637	77.2	19 Aug 20^a^
AL	44.2	39.8	5.6, < .001	6.7	5.2	4.7, < .001	16,376	40.1	5 May 20
AM	49.9	40.8	13.0, < .001	6.2	3.6	9.5, < .001	24,533	50.3	20 Jul 20
AP	48.1	42.0	6.8, < .001	4.9	3.8	3.6, < .001	20,248	50.2	19 Aug 20 ^a^
BA	43.9	40.0	5.4, < .001	2.9	4.0	-6.9, < .001	19,324	27.3	30 Apr 20
CE	48.5	41.3	9.7, < .001	8.3	5.3	7.3, < .001	17,178	28.9	30 May 20
DF	46.9	39.5	10.0, < .001	5.7	3.4	12.8, < .001	85,661	70.0	30 Apr 20
ES	44.8	38.4	7.5, < .001	4.2	3.5	3.7, < .001	34,493	63.1	25 May 20
GO	42.4	36.3	8.6, < .001	7.1	4.8	9.9, < .001	28,273	65.5	19 Apr 20
MA	44.9	38.9	9.2, < .001	5.3	3.5	7.0, < .001	13,956	26.7	23 Apr 20
MG	43.8	37.6	8.2, < .001	5.0	3.1	10.4, < .001	29,223	58.2	19 Aug 20^a^
MS	43.2	38.0	6.6, < .001	2.1	1.7	5.4, < .001	38,926	65.2	22 Jun 20
MT	42.4	38.5	5.4, < .001	4.0	3.4	3.5, < .001	39,931	66.4	22 Apr 20
PA	45.9	39.2	9.0, < .001	5.4	4.3	5.2, < .001	18,952	45.2	14 May 20
PB	44.0	39.4	6.5, < .001	3.5	5.0	-7.0, < .001	16,108	35.0	2 May 20
PE	47.6	40.6	8.4, < .001	6.9	5.5	4.7, < .001	19,624	33.5	16 May 20
PI	44.8	41.1	5.0, < .001	5.8	6.2	-1.4, .165	15,432	23.0	22 Apr 20
PR	45.0	38.1	7.8, < .001	2.9	2.6	2.0, .044	38,773	68.4	28 Apr 20
RJ	49.0	40.4	11.4, < .001	5.1	4.2	4.5, < .001	44,223	68.0	4 Jun 20
RN	42.9	39.1	5.5, < .001	6.0	5.2	2.8, .006	19,250	36.6	7 May 20
RO	45.3	41.0	5.6, < .001	5.4	4.8	2.8, .005	25,554	72.2	14 May 20
RR	42.7	38.7	5.7, < .001	6.3	4.5	6.0, < .001	23,189	72.6	27 May 20
RS	47.5	39.8	7.8, < .001	5.0	5.8	-4.2, < .001	40,363	63.2	10 May 20
SC	47.3	38.4	9.1, < .001	6.4	3.1	14.4, < .001	42,149	75.9	19 Aug 20^a^
SE	42.4	38.3	6.0, < .001	6.0	5.4	2.3, .022	18,443	32.5	7 May 20
SP	46.2	38.7	9.3, < .001	5.6	4.5	5.2, < .001	48,542	68.0	7 May 20
TO	40.2	35.6	6.2, < .001	2.8	3.0	-1.8, .071	22,933	49.0	5 May 20

AC, Acre; AL, Alagoas; AM, Amazonas; AP, Amapá; BA, Bahia; CE, Ceará; DF, Distrito Federal; ES, Espírito Santo; GO, Goiás; MA, Maranhão; MG, Minas Gerais; MS, Mato Grosso do Sul; MT, Mato Grosso; PA, Pará; PB, Paraíba; PE, Pernambuco; PI, Piauí; PR, Paraná; RJ, Rio de Janeiro; RN, Rio Grande do Norte; RO, Rondônia; RR, Roraima; RS, Rio Grande do Sul; SC, Santa Catarina; SE, Sergipe; SP, São Paulo; TO, Tocantins.

^a^ From 19 Aug 2020 on, wearing a face mask in public and private places became mandatory in the whole country (after the National Congress overrode a presidential veto). By then, these states had not yet adopted full mandatory masking.

Table 2 shows the proportion of days in which full, partial or very limited/non-existent restrictions were in force, with information for each of the six different types of social distancing rules in the 27 Brazilian states. Considering the average for Brazil as a whole (last row in Table 2), the data show that full restrictions for industries were the least common type of restriction, and full restrictions on schools and partial restrictions on gatherings were the most common ones. These data are also presented in maps in the supplemental appendices (S3 and S4 Figs).

**Table 2:** Proportion of days in which social distancing rules were in force, by type of restriction and level of strictness (11^th^ March– 10^th^ November 2020), by state, Brazil, 2020.

State	Gatherings, culture, sports, religion (%)			Non-essential shops (%)			Restaurants, bars, etc. (%)			Non-essential industry (%)			Schools (%)			Public transportation (%)		
	0	1	2	0	1	2	0	1	2	0	1	2	0	1	2	0	1	2
AC	2.0	56.3	41.6	38.4	46.9	14.7	3.7	53.5	42.9	100.0	0.0	0.0	2.9	0.0	97.1	44.9	55.1	0.0
AL	0.8	57.6	41.6	22.9	64.9	12.2	22.9	35.5	41.6	97.1	0.0	2.9	4.9	6.5	88.6	22.9	24.9	52.2
AM	2.0	98.0	0.0	65.3	5.7	29.0	38.0	33.1	29.0	100.0	0.0	0.0	2.4	53.1	44.5	3.7	73.5	22.9
AP	2.4	55.5	42.0	59.2	20.0	20.8	52.7	20.0	27.3	100.0	0.0	0.0	2.9	0.0	97.1	89.0	0.0	11.0
BA	2.0	98.0	0.0	65.3	34.7	0.0	58.4	41.6	0.0	100.0	0.0	0.0	2.9	0.4	96.7	21.6	78.4	0.0
CE	2.0	60.8	37.1	34.3	37.1	28.6	4.9	58.0	37.1	34.3	37.1	28.6	2.4	17.6	80.0	34.3	37.1	28.6
DF	0.0	69.0	31.0	65.7	25.7	8.6	49.8	2.0	48.2	100.0	0.0	0.0	0.4	0.0	99.6	100.0	0.0	0.0
ES	2.9	97.1	0.0	23.7	64.9	11.4	25.7	62.9	11.4	100.0	0.0	0.0	4.9	23.7	71.4	100.0	0.0	0.0
GO	0.8	84.9	14.3	56.7	33.5	9.8	7.3	49.4	43.3	61.2	29.0	9.8	2.0	3.7	94.3	3.7	96.3	0.0
MA	2.0	79.2	18.8	4.9	80.8	14.3	60.0	14.3	25.7	94.3	5.7	0.0	2.4	40.8	56.7	75.1	24.9	0.0
MG	2.0	80.0	18.0	84.5	15.5	0.0	84.5	0.0	15.5	100.0	0.0	0.0	2.9	17.1	80.0	6.9	93.1	0.0
MS	3.7	76.3	20.0	100.0	0.0	0.0	100.0	0.0	0.0	100.0	0.0	0.0	4.9	95.1	0.0	100.0	0.0	0.0
MT	2.0	84.5	13.5	86.5	13.5	0.0	33.1	21.6	45.3	100.0	0.0	0.0	4.9	0.0	95.1	95.5	4.5	0.0
PA	2.0	75.5	22.4	4.9	95.1	0.0	4.9	66.5	28.6	92.7	7.3	0.0	2.9	29.0	68.2	64.9	35.1	0.0
PB	4.9	83.7	11.4	4.9	78.0	17.1	4.9	72.2	22.9	100.0	0.0	0.0	3.3	0.0	96.7	89.0	0.4	10.6
PE	2.0	60.8	37.1	16.7	49.0	34.3	16.7	34.7	48.6	100.0	0.0	0.0	2.9	6.5	90.6	4.9	60.8	34.3
PI	2.0	46.5	51.4	4.9	46.5	48.6	4.9	35.1	60.0	95.1	2.9	2.0	2.4	0.4	97.1	25.3	70.2	4.5
PR	2.0	98.0	0.0	49.4	50.6	0.0	94.7	5.3	0.0	100.0	0.0	0.0	3.7	75.9	20.4	49.4	50.6	0.0
RJ	2.4	63.7	33.9	34.3	65.7	0.0	3.3	96.7	0.0	100.0	0.0	0.0	2.9	23.7	73.5	29.4	70.6	0.0
RN	2.9	51.4	45.7	10.2	53.1	36.7	10.2	44.1	45.7	100.0	0.0	0.0	2.9	15.1	82.0	38.4	61.6	0.0
RO	3.7	78.0	18.4	7.3	84.1	8.6	7.3	82.9	9.8	96.7	3.3	0.0	2.4	8.6	89.0	12.2	87.8	0.0
RR	2.4	46.5	51.0	34.3	65.7	0.0	45.3	0.0	54.7	100.0	0.0	0.0	2.4	0.8	96.7	62.9	0.0	37.1
RS	3.7	96.3	0.0	3.7	93.9	2.4	3.7	55.9	40.4	37.6	62.4	0.0	3.3	9.0	87.8	3.7	96.3	0.0
SC	2.4	83.7	13.9	86.1	2.9	11.0	86.1	0.0	13.9	85.3	14.7	0.0	3.3	0.4	96.3	34.7	31.4	33.9
SE	2.4	57.6	40.0	22.9	48.6	28.6	22.4	16.7	60.8	100.0	0.0	0.0	2.9	3.7	93.5	4.9	95.1	0.0
SP	2.0	56.3	41.6	4.9	66.5	28.6	4.9	63.7	31.4	100.0	0.0	0.0	2.0	17.1	80.8	100.0	0.0	0.0
TO	4.9	95.1	0.0	97.1	2.9	0.0	97.1	2.9	0.0	97.1	2.9	0.0	2.0	18.8	79.2	21.6	78.4	0.0
Brazil (average)	2.4	73.7	23.9	40.3	46.1	13.5	35.1	35.9	29.0	92.3	6.1	1.6	3.0	17.3	79.7	43.2	48.1	8.7

AC, Acre; AL, Alagoas; AM, Amazonas; AP, Amapá; BA, Bahia; CE, Ceará; DF, Distrito Federal; ES, Espírito Santo; GO, Goiás; MA, Maranhão; MG, Minas Gerais; MS, Mato Grosso do Sul; MT, Mato Grosso; PA, Pará; PB, Paraíba; PE, Pernambuco; PI, Piauí; PR, Paraná; RJ, Rio de Janeiro; RN, Rio Grande do Norte; RO, Rondônia; RR, Roraima; RS, Rio Grande do Sul; SC, Santa Catarina; SE, Sergipe; SP, São Paulo; TO, Tocantins.

Values are of 0 for non-existent or very limited restrictions, 1 for partial restrictions, and 2 for full restrictions.

In Table 3, results from the regressions indicate that levels of social distancing depend on the strictness of mandatory social distancing rules, their duration, and mandatory use of face masks. Model 1 shows that stricter social distancing rules are associated to increased social distancing–one unit increase in the SDI (in a scale of 0 to 10) is associated with 1.42 percentage point increase in social distancing (*p* < .001, 95% CI [1.25, 1.58]). Duration of rules was also significant, with each additional day of social distancing rules in place decreasing social distancing by -0.015 percentage point (*p* < .001, 95% CI [-0.020, -0.010]). This confirms the hypothesis of ‘pandemic fatigue’, empirically demonstrated in previous research [38], though the effect found in our models was small. Mandatory masking in both public and private spaces reduced social distancing by -3.41 percentage points (*p* < .001, 95% CI [-4.30, -2.51]). The effects of the rates of new COVID-19 cases, GDP per capita, and political orientation of the electorate were not significant in model 1.

**Table 3:** Determinants of social distancing in Brazil. Regression analyses considering six models (11^th^ March– 10^th^ November 2020).

Variables	Model 1		Model 2		Model 3		Model 4		Model 5		Model 6	
	*β*	*p*	*Β*	*p*	*β*	*p*	*β*	*p*	*β*	*p*	*β*	*p*
Social distancing rules index (SDI)	1.416 (0.083)	< .001	1.418 (0.083)	< .001	1.418 (0.083)	< .001	1.275 (0.086)	< .001	1.612 (0.082)	< .001	1.876 (0.107)	< .001
Duration of social distancing rules	-0.015 (0.002)	< .001	-0.015 (0.003)	< .001	-0.015 (0.003)	< .001	-0.029 (0.002)	< .001			0.010 (0.004)	.005
Mandatory use of masks												
*Partial*	-2.282 (0.433)	< .001	-2.245 (0.403)	< .001	-2.249 (0.403)	< .001			-3.009 (0.367)	< .001	-1.707 (0.407)	< .001
*Full*	-3.408 (0.456)	< .001	-3.350 (0.411)	< .001	-3.347 (0.411)	< .001			-5.242 (0.349)	< .001	-2.137 (0.398)	< .001
Incidence rate of new COVID-19 cases	0.004 (0.009)	.657										
Log GDP per capita	0.361 (0.984)	.714	0.711 (0.478)	.137								
Political orientation of voters	0.012 (0.029)	.689			0.019 (0.015)	.218						
SDI × Duration of social distancing rules											-0.009 (0.001)	< .001
Weekend or bank holiday	6.177 (0.242)	< .001	6.175 (0.241)	< .001	6.175 (0.241)	< .001	6.220 (0.243)	< .001	6.137 (0.238)	< .001	6.176 (0.240)	< .001
State dummies	No		No		No		Yes		Yes		Yes	
# of observations	6615		6615		6615		6615		6615		6615	
*R* ^2^	.62		.62		.62		.66		.67		.70	

• Coefficients reported in the table represent social distancing levels in percentage points varying from 0–100. Cluster-robust standard errors between brackets.

In models 2 and 3, first GDP per capita, then political orientation of voters was included. Neither variable was statistically significant in these models. In models 4 and 5, either duration of social distancing rules or mandatory masking was included. Both remained statistically significant, with higher coefficients than in models 1–3, suggesting that each may be in part capturing the effects of the other. The coefficients for the SDI were statistically significant across all models.

In model 6, we kept the significant variables of previous models (*p* < .05) and introduced an interaction between the duration of social distancing rules and their strictness. The effect of mandatory masking remained significant, but less substantial than in the other models.

**Fig 1:**
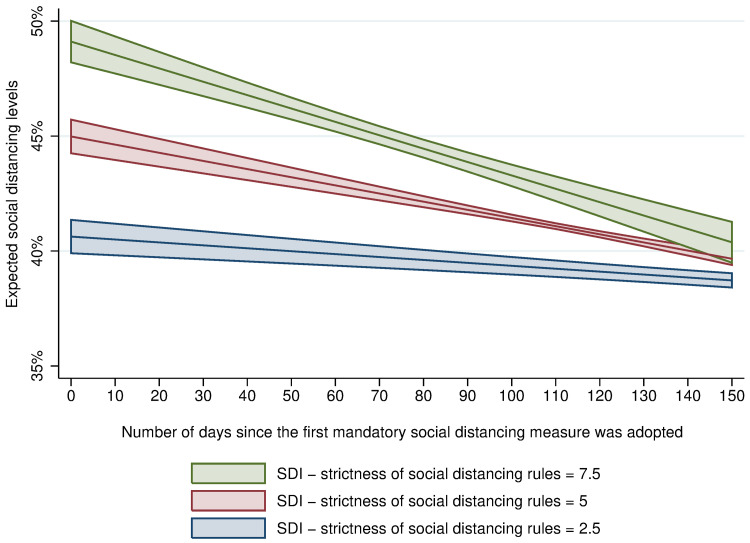
Effect of the duration of mandatory social distancing measures on social distancing levels, by levels of strictness, Brazil (11^th^ March– 10^th^ November 2020). Data show the effect of the duration of social distancing rules on social distancing levels conditioned by the strictness of social distancing rules, and controlling for whether wearing a mask was mandatory (as in model 6, *R*^2^ = 0.70). Shading indicates 95% confidence intervals. The distribution of values of the SDI in the dataset was the following: 0 (2.2%); 0.8 (0.7%); 1.7 (6.3%); 2.5 (15.1%); 3.3 (17.6%); 4.2 (13.5%); 5 (14.5%); 5.8 (8.3%); 6.7 (8.9%); 7.5 (8.3%); 8.3 (2.8%); 9.2 (0.7%); 10 (1.2%).

Results also indicate that the effect of mandatory social distancing rules on social distancing levels decreased faster when rules were stricter, suggesting that people are more likely to tolerate mild rules over long periods of time than very strict ones. This is graphically presented in Fig 1, with each line indicating the expected level of social distancing across three different levels of strictness of social distancing rules over a period of 150 days since mandatory social distancing rules were introduced.

**Fig 2:**
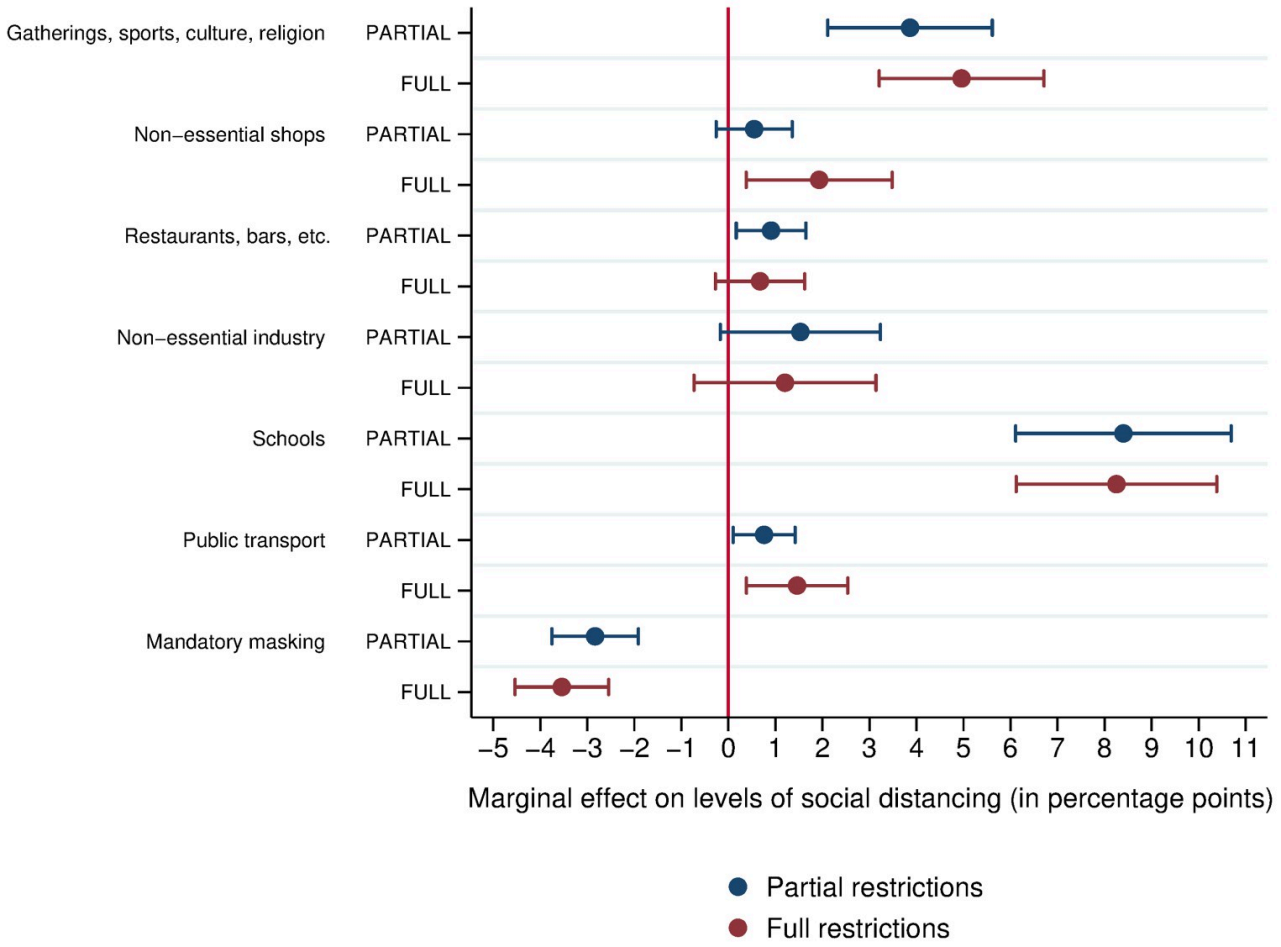
Marginal effect of different types of NPIs on social distancing levels in Brazil (11^th^ March– 10^th^ November 2020), considering partial and full restrictions. Marginal effects of different types of mandatory social distancing rules and mandatory masking on levels of social distancing, controlling for the number of days since measures were introduced, whether wearing a mask was mandatory, and including dummies for each Brazilian state (as in model 7, *R*^2^ = 0.72). Error bars indicate 95% confidence intervals. Coefficients of the other variables are in S2 Table.

None of these models discriminate the individual effects of different NPIs. For example, what is the impact on social distancing levels of suspending in-person classes relative to suspending industrial activities? Does it make sense to close restaurants if this may lead people to go more often to the supermarket? Results from models 7–9 (S2 Table) indicate the effects on social distancing levels of different types of NPIs, with results graphically presented in Fig 2. The effects of closing schools were the strongest. The effects of the following types of restrictions were also significant: partial and full suspension of gatherings, sports, cultural and religious activities; full suspension of non-essential shops; partial suspension of restaurants and bars; and partial and full suspension of public transportation. The following mandatory social distancing rules were significant across models 7–9: partial and full suspension of gatherings, sports, cultural and religious activities; full suspension of non-essential shops; and partial and full suspension of in-person classes (S2 Table).

**Fig 3:**
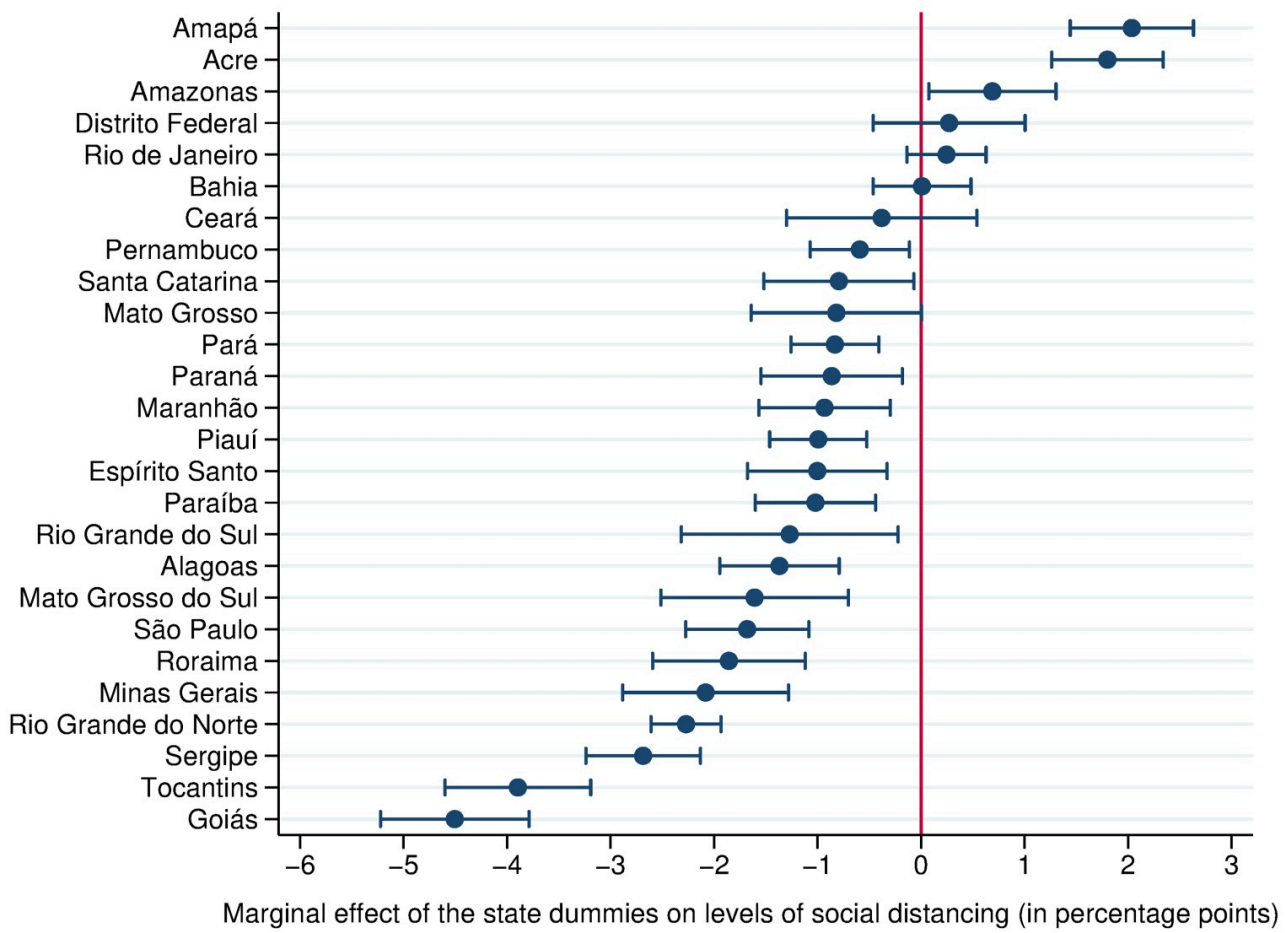
Social distancing levels across different Brazilian states: Estimated coefficients of the state dummies (11^th^ March– 10^th^ November 2020). The state of Rondônia was randomly selected as the state of reference. Coefficients represent the expected difference in social distancing levels relative to the state of Rondônia, controlling for the six different types of restrictions, number of days since the first social distancing measure was introduced and whether wearing a mask was mandatory (as in model 7, *R*^2^ = 0.72). Error bars indicate 95% confidence intervals.

Furthermore, people systematically practiced more social distancing in certain states than in others, even when controlling for other covariates, as shown by the coefficients of the state dummies (Fig 3, from model 7). The coefficients are in percentage points so that, for example, Amazonas had a level of social distancing that was on average around 5 percentage points higher than that of Goiás.

## Discussion

Our study suggests that mandatory social distancing rules have a significant and substantial effect on social distancing levels: the stricter the restrictions the more likely people are to stay at home. The effect of these restrictions is not constant over time though: the longer the rules are in effect the lower the compliance, and the stricter the rules the more compliance with rules decreases over time. Among the restrictions, partial and full suspension of gatherings, cultural, sport and religious activities; full suspension of non-essential shops; and partial and full suspension of schools were significant across different models.

Regarding the severity of the epidemic, an increase in the incidence of new COVID-19 cases did not affect how much people stayed at home. We hypothesize that this may be related to the observation that social distancing levels were higher in the early stages of the pandemic, when people reacted primarily to international incidence and fear, rather than evidence of local disease occurrence. Available evidence suggest that risk perceptions and self-reported protective behaviour were significantly increased in the first weeks of the pandemic [39]. Moreover, the effect of the number of new cases might be different in situations with a much higher number of cases or deaths. In these situations, people would be more likely to experience the direct effects of the pandemic (e.g., relatives or friends getting infected or dying), which might increase their level of concern and, as a consequence, change their behaviour. Results for GDP per capita and political preferences of the electorate were not significant, but these findings should be interpreted with caution as there was no variation in these two variables over time.

Data also show that people were more likely to practice social distancing in certain states than in others (Fig 3). The level of social distancing varied by 7 percentage points across the 27 states due to factors not included in the regressions. This implies that achieving high levels of social distancing in some states requires stricter rules than in others. It is not possible though to infer which features of these states contributed to their higher or lower levels of social distancing. They might be caused by a greater risk aversion among the population of some states, campaigns ran by the state or local governments, or weather patterns, for example.

In summary, the following hypotheses were confirmed:

- the stricter the mandatory social distancing rules the more people stayed at home;
- the stricter the social distancing rules the more compliance decreased over time;
- mandatory masking decreased social distancing levels;
- some types of mandatory social distancing rules had a greater impact on social distancing levels than others. Partial and full suspension of schools and gatherings (alongside sports, cultural and religious activities) were equally effective, indicating that allowing a few selected activities to return is unlikely to have a significant impact on social distancing levels.

This study has a few limitations. First, situations in the category of ‘partial suspension’ may vary across different states, over time, and across different types of restrictions, ranging from more to less restrictions. For example, a few state governments authorized only private schools to reopen (which we considered by definition a case of ‘partial suspension’). Yet, the proportion of students in private schools varies significantly across different states: the lowest in Acre–around 5%–and the highest in Rio de Janeiro–around 31%, implying a potential larger effect on social distancing in the latter [40]. Second, the social distancing rules index (SDI) measures the *legal* ruling of suspension or restriction of activities, but not its enforcement; and places where the enforcement capacity is limited might have lower levels of compliance. Although this is likely to be captured by the state dummy variables, the strength of the effect is unknown. Third, the models do not account for other policies that might also increase social distancing. Awareness campaigns and cash transfers, for example, may increase the likelihood of someone staying at home [25,41–43], and these also varied across different states. Fourth, there is variation over time for some variables of interest, but not for others, either because there was no daily data or because these only change substantially over longer periods of time.

These findings have important policy implications. First, governments must adopt mandatory social distancing rules if they aim at increasing social distancing to high levels, which is especially critical in places with low capacity for case detection and contact-tracing [5]. Second, stricter rules increase social distancing levels more than mild ones, but compliance decreases proportionally more over time when rules are stricter. The timing for adopting strict rules is therefore important because compliance may decrease when most needed. This problem may be partially solved by an on-off lockdown policy [44]. Third, policymakers should invest in more targeted policies. Suspending in-person classes, gatherings, religious, sport and cultural activities, for example, increases more social distancing than suspending industrial activities.

Social distancing, however, is obviously not an end in itself, but a means to reduce COVID-19 cases and deaths, and it is important to learn more about which forms of social distancing are most effective against cases and deaths. Policy makers will want to weigh the burdens imposed by social distancing measures against their effects on cases and deaths. Closing schools, for example, has a large effect on levels of social distancing, but also may have large negative impacts on children and their parents–especially single parents and children without access to the Internet–so that it is important to know its effects on cases and deaths in order to be able to weigh its positive effects against the negative impacts. It may also be possible to identify modifications of some social distancing measures that reduce the burdens: for example, people are more likely to maintain social distancing in an assembly line than in a mass gathering, so policy-makers should consider suspending gatherings for a longer period of time than other types of activities. And masking, which reduces the need for social distancing, is effective with much less burden on social and economic activities.

Finally, although widespread vaccination is reducing the severity of the pandemic, social distancing measures remain an effective strategy to reduce transmission, even if not necessarily with the same level of strictness of earlier stages of the pandemic. Moreover, strict social distancing rules should be once more adopted in case vaccines prove less effective against new variants or, of course, if other large epidemics emerge in the future.

## Supporting information

S1 Fig Strictness of social distancing measures and levels of social distancing (11^th^ March– 10^th^ November 2020), centred-moving averages (seven days). (TIF)

S2 Fig Strictness of social distancing measures and social distancing levels, Brazilian states (11^th^ March– 10^th^ November 2020). (TIF)

S3 Fig Percentage of days in which there were full restrictions on social or economic activities, Brazilian states (11^th^ March– 10^th^ November 2020). (TIF)

S4 Fig Percentage of days in which there were partial or full restrictions on social or economic activities, Brazilian states (11^th^ March– 10^th^ November 2020). (TIF)

S1 Table Criteria for coding the strictness of social distancing rules (by level of strictness). (DOCX)

S2 Table Determinants of social distancing in Brazil (11^th^ March– 10^th^ November 2020). (DOCX)
