# Correction: Effects of non-pharmaceutical interventions on social distancing during the COVID-19 pandemic: Evidence from the 27 Brazilian states

**DOI:** 10.1371/journal.pone.0314374

**Published:** 2024-11-20

**Authors:** Rodrigo Fracalossi de Moraes, Louise B. Russell, Lara Livia Santos da Silva, Cristiana M. Toscano

There is an error in the S1 and S2 Tables. There are many pages presented of which only the first two pages actually match the title. Please see the correct S1 and S2 Tables here.
