# Supplementary figures and images for: Effects of non-pharmaceutical interventions on social distancing during the COVID-19 pandemic: Evidence from the 27 Brazilian states
Source: PLoS One. 2022 Mar 17;17(3):e0265346. doi: 10.1371/journal.pone.0265346 (PMC8929638; doi:10.1371/journal.pone.0265346)

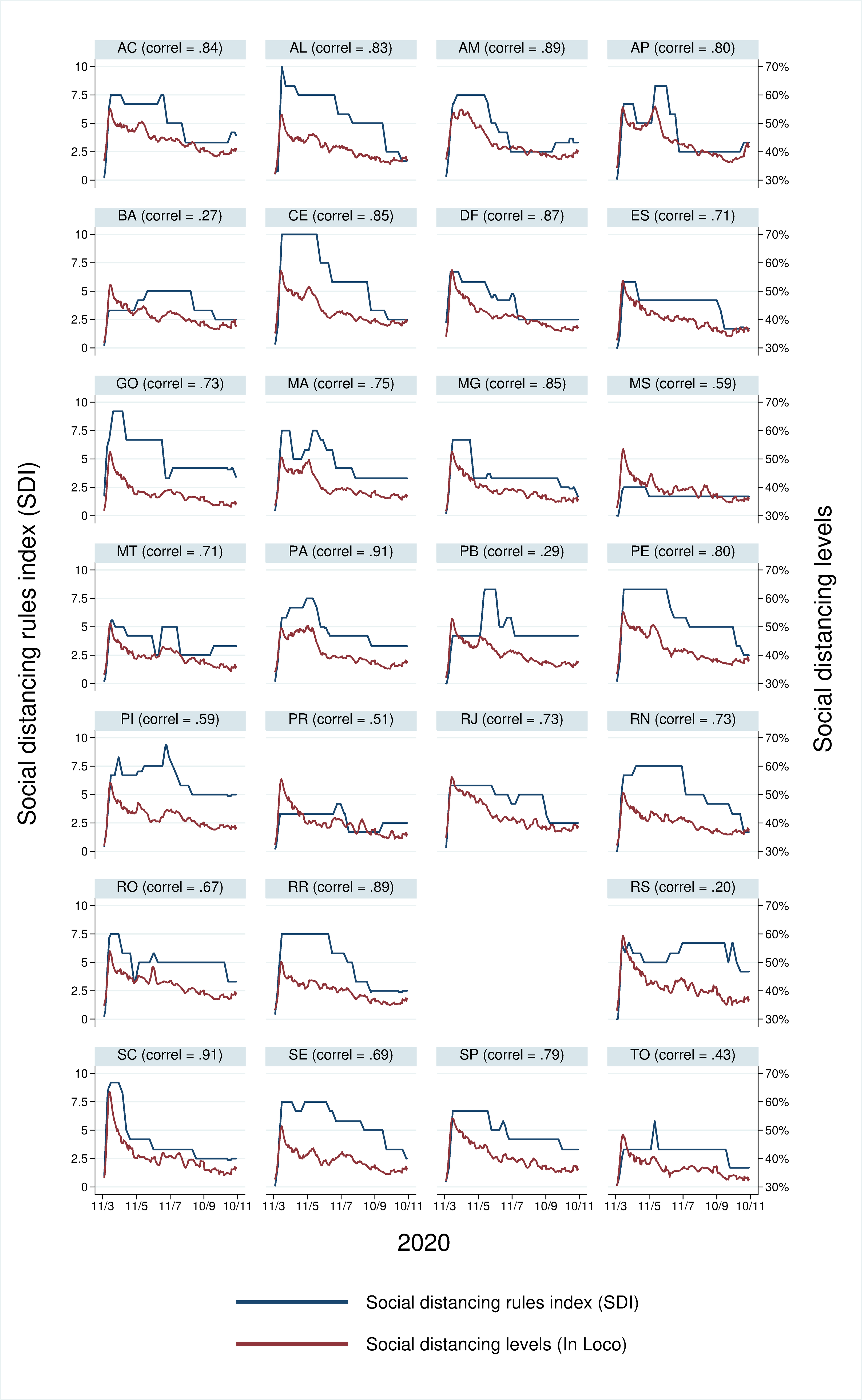

Supplement: S1 Fig — (TIF) [file pone.0265346.s001.tif]

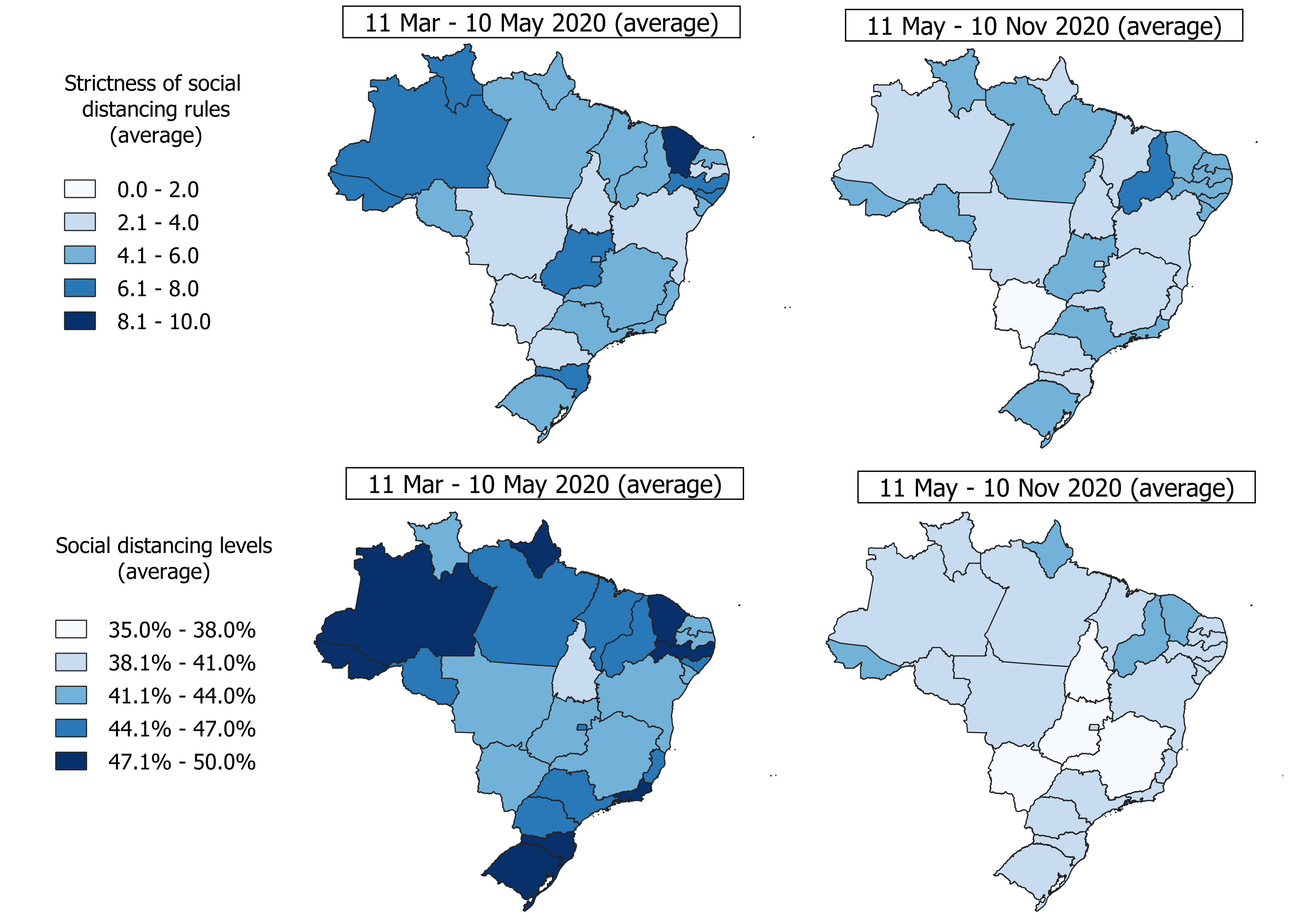

Supplement: S2 Fig — (TIF) [file pone.0265346.s002.tif]

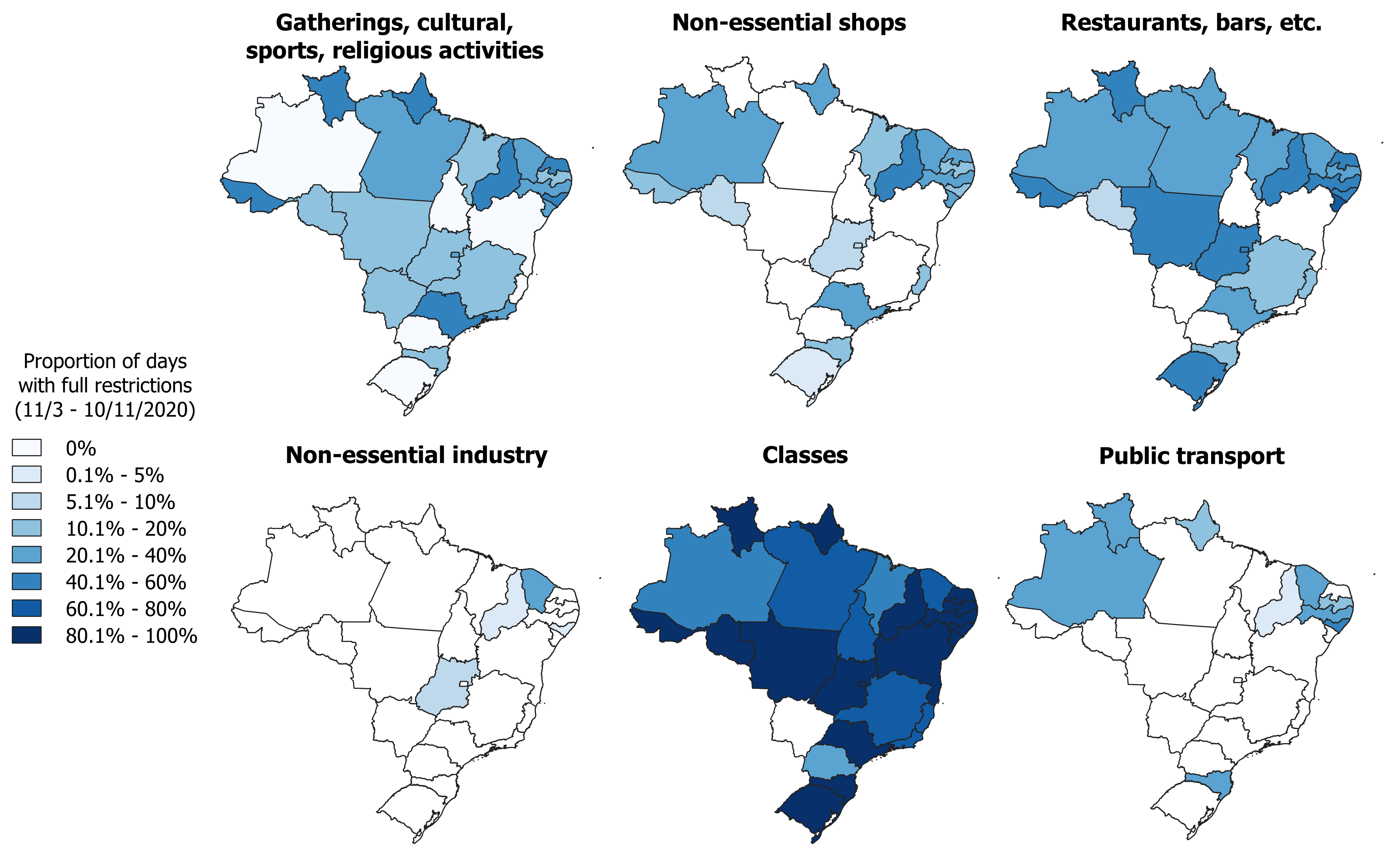

Supplement: S3 Fig — (TIF) [file pone.0265346.s003.tif]

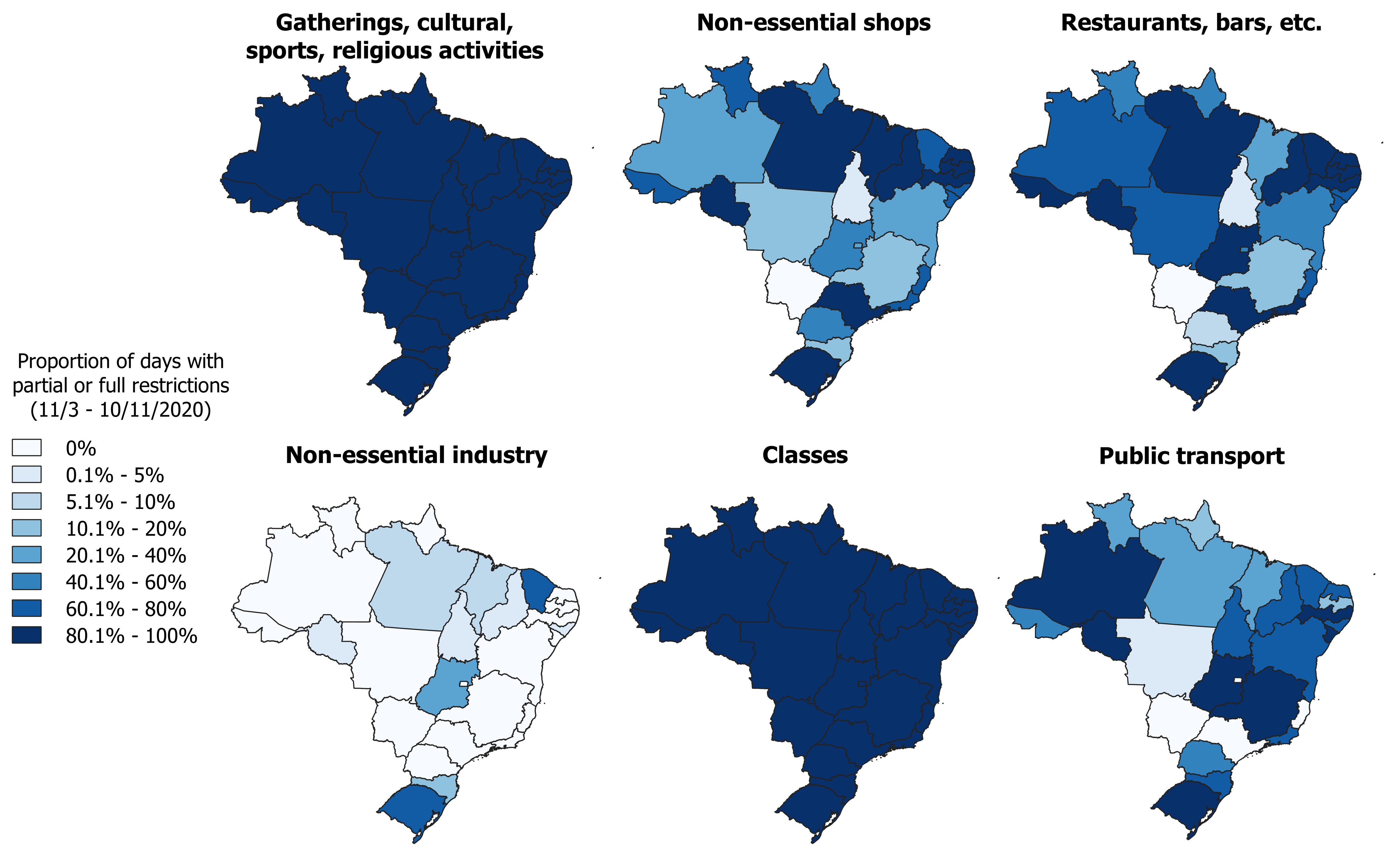

Supplement: S4 Fig — (TIF) [file pone.0265346.s004.tif]
