# Supplementary material for: Effects of non-pharmaceutical interventions on social distancing during the COVID-19 pandemic: Evidence from the 27 Brazilian states
Source: PLoS One. 2022 Mar 17;17(3):e0265346. doi: 10.1371/journal.pone.0265346 (PMC8929638; doi:10.1371/journal.pone.0265346)
Supplement: S1 Table — (DOCX) [file pone.0265346.s005.docx]

**S1 Table. Criteria for coding the strictness of social distancing rules (by level of strictness).**

| **Social distancing measure** | **Level of strictness** | | |
| --- | --- | --- | --- |
|  | **2**  **(Full suspension/closure)** | **1**  **(Partial suspension/closure)** | **0**  **(Very limited or no suspension)** |
| **Gatherings (private and public) and cultural, sport or religious activities** | Gatherings with more than 20 people are prohibited. In person activities at the following places are suspended: gyms, places of worship, concert halls, cinemas, theatres, cultural centres, museums, libraries, and art galleries. | One or a few of these activities or places is suspended or closed (even if only in part of the state’s territory).  Examples: places of worship can open, but mass gatherings are prohibited; cinemas and theatres are open but concert halls are not; large musical concerts are prohibited but cinemas are open. | There are no restrictions or they are very limited.  Examples: Gyms can open but must check people’s temperature; cinemas can use 80% of their capacity;  gatherings with fewer than 2000 people are authorized. |
| **Non-essential shops and offices** | All non-essential shops and offices (e.g., furniture stores and shopping centres) must remain closed except for delivery or takeaway. | Only one or a few types of non-essential shops or offices are closed (even if only in part of the state´s territory) or are allowed to open, but at no more than 50% capacity.  Examples: Department stores and shopping centres are closed but other shops are open; furniture and clothing stores are open but beauty salons are not. | There are no restrictions or they are very limited.  Examples: Shopping centres are allowed to open but must close at 9pm. |
| **Bars, pubs, restaurants and similar places** | The following places cannot open: bars, pubs, restaurants, cafés and similar places, except for delivery or takeaway. | One or a few of these activities or places is suspended or closed (even if only in part of the state’s territory), or there are strict rules if they are open.  Examples: Restaurants can open but bars/pubs are closed; restaurants, pubs and bars can open only for lunch; restaurants are open but at no more than 50% capacity. | There are no restrictions or they are very limited.  Examples: All restaurants are allowed to open but must close at 10pm; bars and pubs can open but live music is not allowed. |
| **Non-essential industry** | All non-essential industrial activities are suspended. | One or a few non-essential industrial activities are suspended (even if only in part of the state´s territory) or industrial activities are allowed, but at no more than 50% capacity.  Examples: All industries are authorized to open but at no more than 50% capacity; textile industry can fully operate but the furniture industry cannot. | There are no restrictions or they are very limited.  Examples: Industrial units are authorized to open but at no more than 80% capacity. |
| **Schools ^a^** | In person activities at schools and universities are not allowed | Some schools or universities can open (even if in part of the state’s territory) and/or they are allowed to open at no more than 50% capacity.  Examples: Only primary schools are open; only universities are open; all schools and universities are open, but at no more than 50% capacity. | There are no restrictions or they are very limited.  Examples: Schools can open at no more than 90% capacity; only students at the last year of secondary school or at public kindergartens can attend classes. |
| **Public transportation ^b^** | Intermunicipal and interstate public transport are suspended, except for the transportation of essential workers (health workers, for example). | Only intermunicipal *or* interstate public transport is suspended, or vehicles can circulate but at no more than 50% capacity (even if only in part of the state´s territory). | There are no restrictions or they are very limited.  Examples: Vehicles can circulate at up to 80% capacity; buses must keep windows open. |
| **Mask mandate** | Mandatory use of face masks in all public and private places.  Examples: Use everywhere except at home and in private cars; mandatory use everywhere except in parks. | Mandatory use of face masks in certain places or under certain circumstances.  Examples of partial suspension: mandatory use only in public transportation; only in closed places; for staff but not customers; in some municipalities, but not in others. | Use of face masks is voluntary, or is mandatory only for people who usually wear face masks (healthcare workers), for very specific groups of people or for a few small municipalities. |

^a^ Not including schools and universities run by local (municipal) governments or the federal government.


^b^ Not including local (municipal) transportation.
