# Supplementary material for: Effects of non-pharmaceutical interventions on social distancing during the COVID-19 pandemic: Evidence from the 27 Brazilian states
Source: PLoS One. 2022 Mar 17;17(3):e0265346. doi: 10.1371/journal.pone.0265346 (PMC8929638; doi:10.1371/journal.pone.0265346)
Supplement: S2 Table — (DOCX) [file pone.0265346.s006.docx]

**S2 Table. Determinants of social distancing in Brazil (11^th^ March – 10^th^ November 2020).**

| Variables | Model 7 | | Model 8 | | Model 9 | |
| --- | --- | --- | --- | --- | --- | --- |
|  | *β* | *p* | *β* | *p* | *β* | *p* |
| Gatherings |  |  |  |  |  |  |
| *Partial suspension* | 3.864  (0.892) | < .001 | 3.691  (0.943) | < .001 | 2.939  (0.924) | .001 |
| *Full suspension* | 4.958  (0.894) | < .001 | 4.816  (1.025) | < .001 | 4.850  (0.998) | < .001 |
| Non-essential shops |  |  |  |  |  |  |
| *Partial suspension* | 0.549  (0.412) | .183 | 0.452  (0.448) | .313 | 1.217  (0.479) | .011 |
| *Full suspension* | 1.930  (0.792) | .015 | 2.496  (0.994) | .012 | 2.493  (0.807) | .002 |
| Restaurants, bars |  |  |  |  |  |  |
| *Partial suspension* | 0.908  (0.380) | .016 | 0.714  (0.391) | .067 | 0.718  (0.508) | .157 |
| *Full suspension* | 0.673  (0.484) | .164 | 0.066  (0.554) | .905 | 1.329  (0.572) | .020 |
| Non-essential industry |  |  |  |  |  |  |
| *Partial suspension* | 1.532  (0.868) | .078 | 1.430  (1.048) | .172 | 1.843  (0.869) | .034 |
| *Full suspension* | 1.204  (0.988) | .223 | 1.749  (0.997) | .079 | 0.333  (1.003) | .740 |
| Schools |  |  |  |  |  |  |
| *Partial suspension* | 8.400  (1.169) | < .001 | 7.970  (1.158) | < .001 | 5.890  (1.258) | < .001 |
| *Full suspension* | 8.253  (1.089) | < .001 | 7.424  (1.093) | < .001 | 6.908  (1.082) | < .001 |
| Public transport |  |  |  |  |  |  |
| *Partial suspension* | 0.760  (0.336) | .024 | 0.330  (0.435) | .448 | 1.342  (0.411) | .001 |
| *Full suspension* | 1.461  (0.550) | .008 | 0.975  (0.674) | .148 | 2.068  (0.610) | .001 |
| Duration of social distancing rules | -0.030  (0.003) | < .001 | -0.043  (0.003) | < .001 |  |  |
| Mandatory use of masks |  |  |  |  |  |  |
| *Partial* | -2.834  (0.469) | < .001 |  |  | -3.903  (0.429) | < .001 |
| *Full* | -3.541  (0.507) | < .001 |  |  | -6.513  (0.511) | < .001 |
| Weekend or bank holiday | -6.202  (0.240) | < .001 | -6.241  (0.243) | < .001 | -6.132  (0.236) | < .001 |
| State dummies | Yes | | Yes | | Yes | |
| # of observations | 6615 | | 6615 | | 6615 | |
| *R*² | .72 | | .70 | | .69 | |

Coefficients reported in this table represent social distancing levels in percentage points varying from 0-100. Cluster-robust standard errors between brackets.
